# Structural basis for the potent and selective binding of LDN-212854 to the BMP receptor kinase ALK2

**DOI:** 10.1016/j.bone.2017.09.004

**Published:** 2018-04

**Authors:** Eleanor Williams, Alex N. Bullock

**Affiliations:** Structural Genomics Consortium, University of Oxford, Roosevelt Drive, Oxford OX3 7DQ, UK

**Keywords:** BMP, Heterotopic ossification, Kinase inhibitor, Crystal structure, Drug, Fibrodysplasia ossificans progressiva

## Abstract

Individuals with the rare developmental disorder fibrodysplasia ossificans progressiva (FOP) experience disabling heterotopic ossification caused by a gain of function mutation in the intracellular region of the BMP type I receptor kinase ALK2, encoded by the gene *ACVR1*. Small molecule BMP type I receptor inhibitors that block this ossification in FOP mouse models have been derived from the pyrazolo[1,5-*a*]pyrimidine scaffold of dorsomorphin. While the first derivative LDN-193189 exhibited pan inhibition of BMP receptors, the more recent compound LDN-212854 has shown increased selectivity for ALK2. Here we solved the crystal structure of ALK2 in complex with LDN-212854 to define how its binding interactions compare to previously reported BMP and TGFβ receptor inhibitors. LDN-212854 bound to the kinase hinge region as a typical type I ATP-competitive inhibitor with a single hydrogen bond to ALK2 His286. Specificity arising from the 5-quinoline moiety was associated with a distinct pattern of water-mediated hydrogen bonds involving Lys235 and Glu248 in the inactive conformation favoured by ALK2. The structure of this complex provides a template for the design of future ALK2 inhibitors under development for the treatment of FOP and other related conditions of heterotopic ossification.

## Highlights

• We solved a crystal structure of the BMP receptor ALK2 genetically linked to FOP.
• The inhibitor LDN-212854 bound to the ATP-binding pocket of the kinase domain.
• Binding of the inhibitor was supported by water-mediated hydrogen bonds.
• This hydrogen bonding pattern contributes to the inhibitor's selectivity for ALK2.

## Graphical abstract

**Figure:**
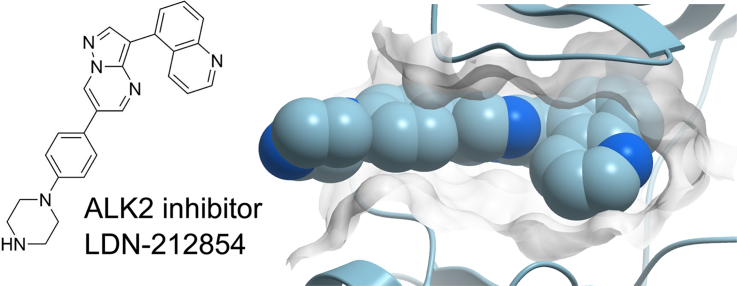

## 1 Introduction

Bone morphogenetic proteins (BMPs) represent the largest subgroup in the TGFβ family of extracellular ligands [1], [2]. Signal transduction by these ligands requires type I and type II transmembrane receptor serine/threonine kinases which form heterotetrameric complexes for ligand interaction. The identity of the ligand determines the specific recruitment of receptors and the downstream signalling [3]. Type I receptors phosphorylate SMAD family transcription factors, whereas type II receptors are required to activate the type I receptors by phosphorylation of their juxtamembrane GS (glycine-serine rich) domain. Type I receptors ALK1/*ACVRL1*, ALK2/*ACVR1*, ALK3/*BMPR1A* and ALK6/*BMPR1B* all participate in BMP signalling and phosphorylate SMAD1/5/8. In contrast, the Activin type I receptors ALK4/*ACVR1B* and ALK7/*ACVR1C* and the TGFβ type I receptor (ALK5/*TGFBRI*) all primarily signal through SMAD2/3. In addition, the receptor signalling complexes can activate a variety of non-canonical pathways, including the p38 MAPK and phosphoinositide 3-kinase (PI3K) signalling cascades. Characterisation of these receptors and their signalling pathways has been pivotal to our understanding of the effects of BMP signalling in processes such as embryonic patterning, tissue differentiation and homeostasis [4], [5], [6].

The importance of BMP signalling is evident from the many disease conditions that are genetically linked to the dysregulation of these pathways [6], [7]. Most commonly these involve loss of function mutations, as exemplified by *ACVRL1* and *BMPR1A* mutations which predispose to hereditary haemorrhagic telangiectasia [8] and juvenile polyposis syndrome [9], [10], respectively. By contrast, fibrodysplasia ossificans progressiva (FOP) is a rare monogenic condition in which a gain of function germline mutation in *ACVR1* leads to increased signalling through ALK2 in response to BMP ligands as well as neofunction in response to Activin A and the consequent formation of heterotopic bone in muscle and connective tissue [11], [12]. Similar somatic mutations in *ACVR1* are also observed in 25% of cases of diffuse intrinsic pontine glioma (DIPG), a rare childhood brain tumour [13]. BMP signalling has also been linked to other human cancers. For example, BMP2 can promote the expansion of ovarian cancer stem cells [14], while BMP6 overexpression is associated with prostate cancer skeletal metastases [15], [16]. Notably, BMP type I receptor inhibitors have demonstrated promising effects in DIPG patient cell lines [17], as well as a number of other cancer models [18], [19], [20], [21], [22], [23], [24], [25]. BMP signalling has also been identified as a promising therapeutic target to normalize hepcidin expression in chronic anaemia of inflammation [26], [27].

**Fig. 1:**
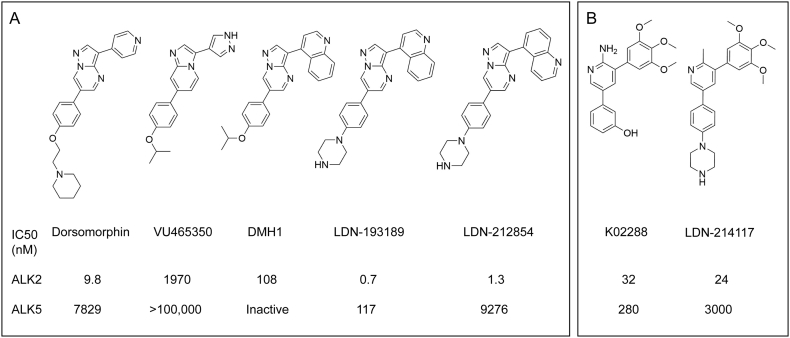
Chemical scaffolds of selected BMP type I receptor inhibitors. (A) Selected inhibitors derived from the core pyrazolo[1,5-*a*]pyrimidine scaffold of dorsomorphin. (B) Inhibitors based on an alternative pyridine-based scaffold. IC_50_ data are those reviewed by Hopkins [38] except the data for LDN-214117 which are taken from Mohedas et al. [37].

These data have stimulated interest in the development of small molecule BMP type I receptor inhibitors both as therapeutic agents and as chemical tools to probe cellular signalling mechanisms [7], [28]. Dorsomorphin was discovered as the first small molecule BMP receptor inhibitor using a phenotypic screen to identify compounds capable of inducing the dorsalization of zebrafish embryos, as observed for the mutant BMP receptor *lost-a-fin* [26]. A crystal structure of the human orthologue ALK2 confirmed the direct binding of dorsomorphin to the ATP-binding pocket of the receptor's intracellular kinase domain [29]. Further development of the pyrazolo[1,5-*a*]pyrimidine-containing scaffold has subsequently produced a series of derivative compounds, including LDN-193189 [30], DMH1 [31], VU465350 [32], and LDN-212854 [33] (Fig. 1a). LDN-193189 was selected for its improved potency and pharmacokinetic properties [30], which have enabled its use in animal models of heterotopic ossification [34], vascular calcification [35] and anaemia of inflammation [27]. DMH1 was identified from further zebrafish screening to reduce off-target activity against the VEGF pathway [31]. Finally, VU465350 and LDN-212854 were recently reported as inhibitors harboring increased subfamily selectivity for ALK3 and ALK2, respectively [32], [33]. In addition, a second series of pyridine-based small molecule BMP type I receptor inhibitors was identified from a biochemical screen against the purified ALK2 kinase domain (Fig. 1b). The initial screening hit K02288 showed remarkable selectivity for BMP receptors over a panel of some 250 human kinases [36]. Subsequent work yielded the compound LDN-214117, which displayed superior activity in cells, as well as enhanced selectivity for ALK2 over ALK5 [37].

The compound LDN-212854 is of particular interest for the development of inhibitors against heterotopic ossification. It has demonstrated notable efficacy in two different mouse models, including one using an inducible constitutively-active *ACVR1*^Q207D^ transgene [33] and another harboring an *Acvr1*^R206H^ knock-in allele that more faithfully recapitulates human FOP [39]. LDN-212854 was developed from LDN-193189 through the substitution of a 4-quinoline moiety for a 5-quinoline (Fig. 1a) [33]. Remarkably, this simple change increased the compound's selectivity for ALK2 over ALK3 from 21-fold (LDN-193189) to 66-fold (LDN-212854). In addition, the selectivity for ALK2 over ALK5 was increased from 175-fold to over 9000-fold [33]. Thus, the ALK2 bias of LDN-212854 is ideally suited to counter the *ACVR1*^R206H^ mutation that underlies FOP. Here we determined the crystal structure of ALK2 in complex with LDN-212854 revealing subtle differences in its binding compared to LDN-193189.

## 2 Materials and methods

### 2.1 Protein expression and purification

The recombinant ALK2 kinase domain was prepared with a Q207D mutation as previously described [36]. Briefly, ALK2 residues 201–499 were cloned into transfer vector pFB-LIC-Bse and baculovirus prepared in DH10Bac cells. Baculoviral expression was performed in Sf9 insect cells grown at 27 °C. Some 48 h post-infection, cells were harvested and lysed using ultrasonication. ALK2 protein was purified sequentially by nickel affinity and size-exclusion chromatography. The eluted protein was stored at − 80 °C buffered in 50 mM HEPES, pH 7.5, 300 mM NaCl, 2 mM DTT, 50 mM arginine, 50 mM glutamate. The N-terminal hexahistidine tag used for purification was cleaved using tobacco etch virus (TEV) protease.

### 2.2 Crystallization

Crystallization was achieved at 4 °C using the sitting-drop vapor diffusion method. ALK2 was preincubated with 1 mM LDN-212854 at a protein concentration of 13.6 mg/mL and crystallized using a precipitant containing 18% PEG8000, 0.2 M calcium acetate, 0.1 M cacodylate pH 6.5. Viable crystals were obtained when the protein solution was mixed with the reservoir solution at 2:1 volume ratio. Crystals were cryoprotected with mother liquor plus 25% ethylene glycol, prior to vitrification in liquid nitrogen.

### 2.3 Data collection

Diffraction data were collected at the Diamond Light Source, station I03 using monochromatic radiation at wavelength 0.97625 Å.

### 2.4 Phasing, model building, refinement, and validation

Data were processed with MOSFLM [40] and subsequently scaled using the program AIMLESS from the CCP4 suite [41]. Initial phases were obtained by molecular replacement using the program PHASER [42] and the structure of ALK2 (PDB ID 3H9R) as a search model. The resulting structure solution was refined using REFMAC5 from the CCP4 suite [43] and manually rebuilt with COOT [44]. Appropriate TLS restrained refinement using the tls tensor files calculated from the program TLSMD [45] was applied at the final round of refinement. The complete structure was verified for geometric correctness with MolProbity [46]. Data collection and refinement statistics are shown in Table 1.

**Table 1:** Data collection and refinement statistics (molecular replacement).

	ALK2-LDN-212854 (PDB ID 5OXG)
*Data collection*	
Space group	*I*121
Cell dimensions	
*a*, *b*, *c* (Å)	85.9, 102.2, 177.3
α, β, γ (°)	90.0, 94.0, 90.0,
Resolution (Å)^a^	88.49–1.73 (1.80–1.73)
*R*_merge_^a^	0.0681 (1.284)
*I*/σ*I*^a^	4.91 (0.56)
Completeness (%)^a^	99.30 (97.95)
Redundancy^a^	1.9 (1.8)
*Refinement*	
Resolution (Å)	2.13
No. reflections^a^	293,366 (27506)
*R*_work_/*R*_free_	0.22/0.25
No. atoms	9810
Protein	9326
Ligand/ion	139
Water	345
B-factors	46.00
Protein	45.90
Ligand/ion	47.10
Water	46.70
R.m.s deviations	
Bond lengths (Å)	0.012
Bond angles (°)	1.49

Data from a single crystal.

a Highest resolution shell is shown in parenthesis.

## 3 Results and discussion

To date, all small molecule BMP receptor inhibitors have been targeted to the ATP-binding pocket located within the intracellular kinase domain of the receptors. While this region is highly conserved across the BMP receptor family, crystallographic studies can reveal small sequence and conformational differences that may be exploited for the design of inhibitor potency and selectivity [29], [36], [37], [47], [48], [49].

### 3.1 Structure determination of the ALK2-LDN-212854 complex

To facilitate structural studies with LDN-212854, we recombinantly expressed the human ALK2 kinase domain in Sf9 insect cells and purified the resulting protein to homogeneity using Ni-affinity and size exclusion chromatography. This protein construct lacks the more flexible GS domain region and has been found to be highly amenable to crystallization. When mixed together, the ALK2-LDN-212854 complex crystallized in sitting drops in space group *I*121 and yielded excellent diffraction allowing structure refinement at 1.73 Å resolution (see Table 1 for diffraction data collection and refinement statistics). Four protein-inhibitor complexes were observed in the asymmetric unit with no significant structural differences.

### 3.2 ALK2 exhibits an inactive kinase conformation

**Fig. 2:**
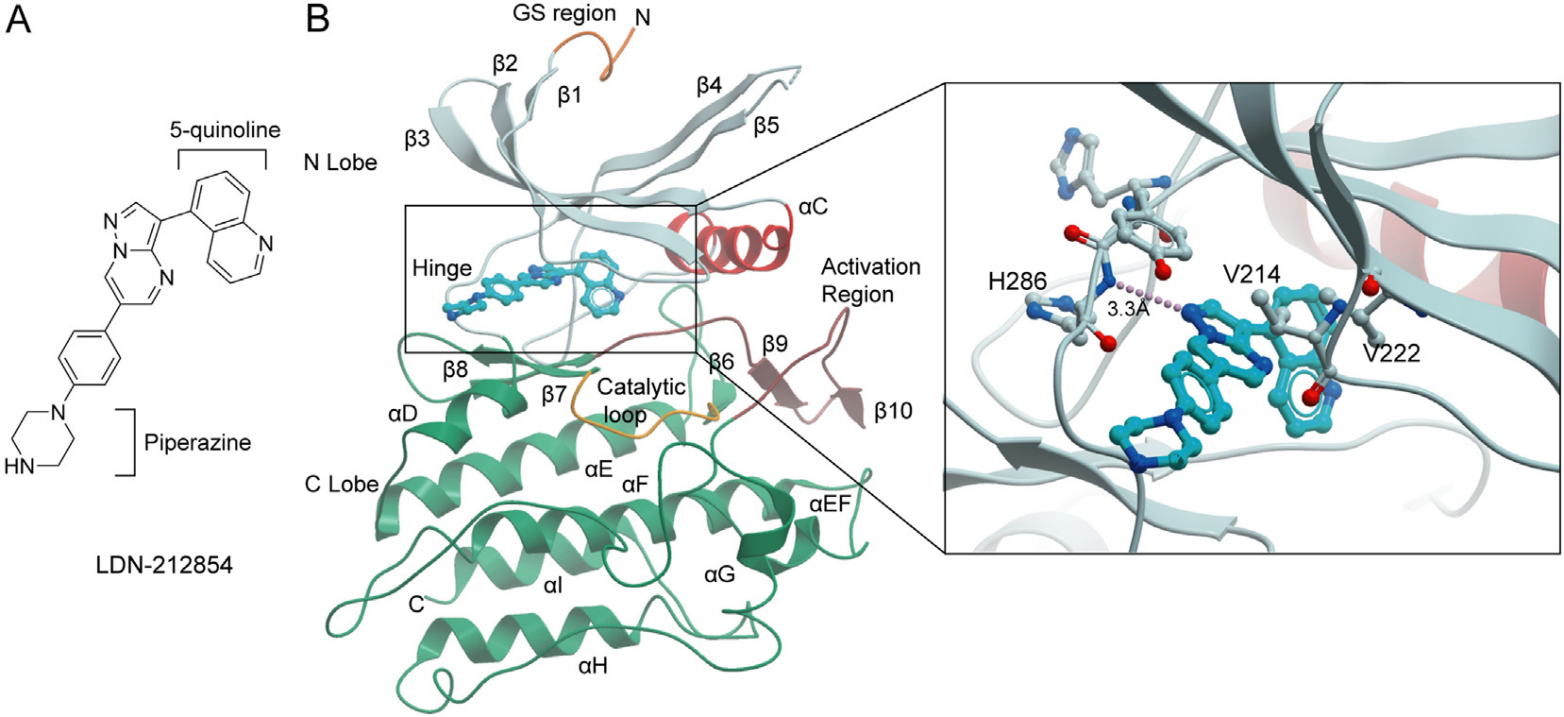
Structure of ALK2 bound to LDN-212854. (A) Chemical structure of LDN-212854 highlighting the piperazine and 5-quinoline moieties. (B) (Left) Ribbon diagram showing the structure of the ALK2 kinase domain. The activation segment (pink) adopts an inactive conformation that inserts into the front of the ATP-binding pocket and would block ATP binding. (Right inset) The compound LDN-212854 binds to the hinge region which connects the N and C-terminal lobes of the kinase domain. The N-1 nitrogen from the core pyrazolo[1,5-*a*]pyrimidine group forms a single hydrogen bond (dashed line) to the backbone amide of H286.

The structure of the ALK2 kinase domain in complex with LDN-212854 was observed to adopt an inactive conformation (Fig. 2) similar to the more complete structure of the ALK2 GS and kinase domains bound to FKBP12 [29]. In this shared inhibitory conformation there is close packing of the kinase N and C-lobes creating strong interactions for the inhibitor bound in the central ATP-binding pocket. However, at the solvent-exposed entrance of the active site the binding of ATP and substrate would be sterically occluded by the inward folding of the activation segment [29]. Importantly, FOP-causative mutations such as R375P disrupt this inhibitory packing and therefore sensitize the mutant ALK2 receptor to activation [29].

### 3.3 Conserved binding mode of LDN-212854

LDN-212854 contains the same core pyrazolo[1,5-*a*]pyrimidine scaffold as LDN-193189, which has been co-crystallized previously with ALK2 [36]. As expected, this group binds to the hinge region which connects the N and C-lobes of the kinase domain (Fig. 2). Here, a single hydrogen bond is observed between the N-1 nitrogen of the pyrazolo[1,5-*a*]pyrimidine moiety and the backbone nitrogen of His286 (Fig. 2). The pendant phenyl ring is also similarly packed below the β1 strand where its hydrophobic interactions include van der Waals contacts with Val214 (Fig. 2). The piperazine ring is largely solvent exposed at the front of the ATP-binding pocket (Fig. 2) and therefore suitable for any future chemical modifications. Most importantly, the central pyrazolo[1,5-*a*]pyrimidine moiety orients the 5-quinoline group to the back pocket where it packs below Val222 (Fig. 2). This region of the ATP-binding pocket presents a richly hydrophobic environment including Val222, Ala232, Leu263, Leu281, Leu343 and Ala353. The large 5-quinoline group exploits this back pocket region similarly to the 4-quinoline of LDN-193189.

### 3.4 Differences in the binding of LDN-212854 and LDN-193189

**Fig. 3:**
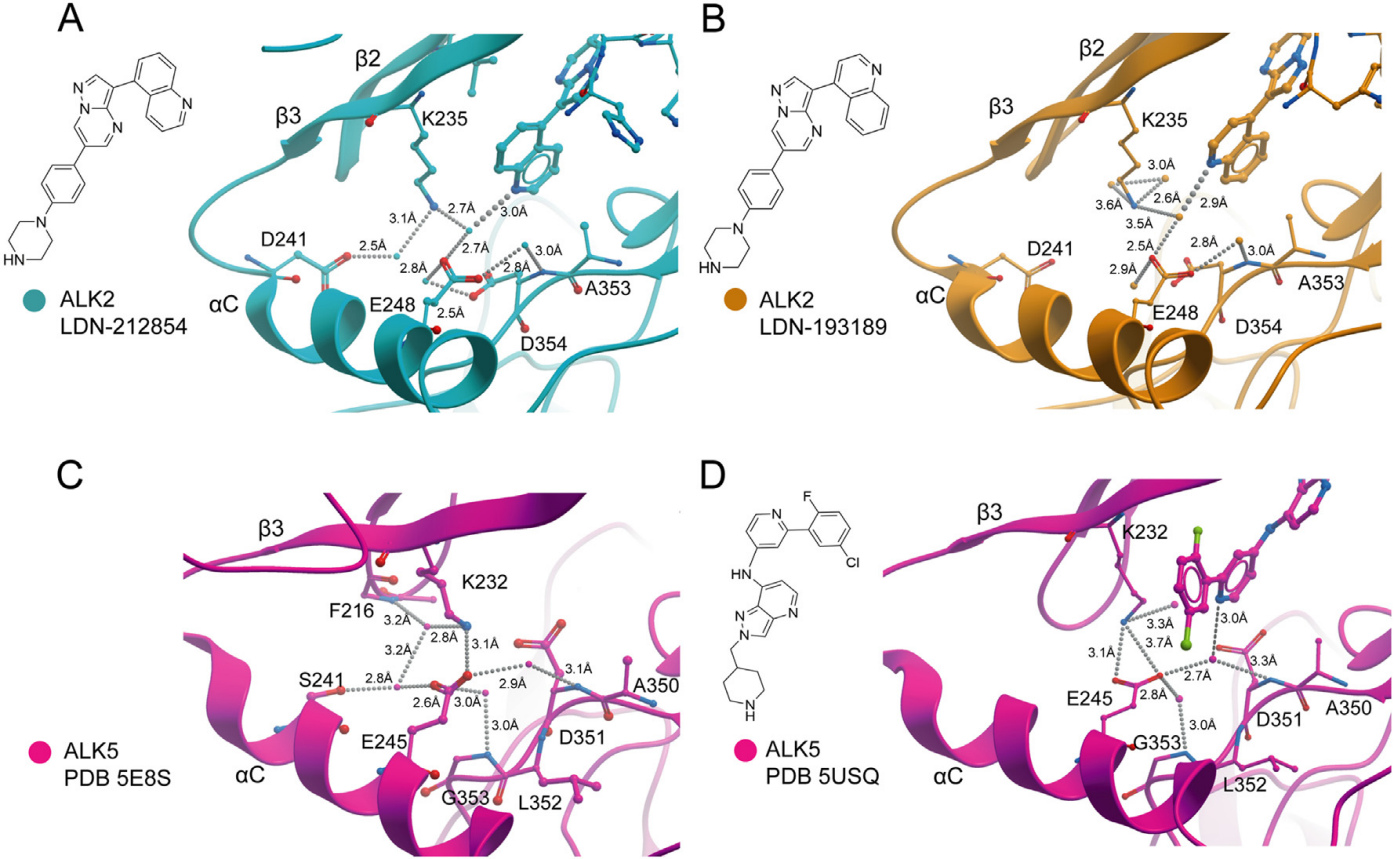
Water-mediated hydrogen bond interactions of selected kinase inhibitor complexes. (A) Close up of the ATP-binding pocket of ALK2 viewed from the side of the αC helix. Hydrogen bonds (dashed lines) are shown to water molecules (single blue spheres) with distances. The 5-quinoline of LDN-212854 forms water-mediated hydrogen bonds with K235 and E248. Other water-mediated interactions are similarly labelled. (B) The 4-quinoline of LDN-193189 forms a water-mediated hydrogen bond to E248, but is too distant (3.54 Å) to contact K235. (C) The apo structure of ALK5 (PDB 5E8S) also shows a water network in this vicinity, but the equivalent residues K232 and E245 side chains are sufficiently close to form a direct salt bridge. (D) An inhibitor bound structure of ALK5 (PDB 5USQ) shows a water-mediated hydrogen bond from the inhibitor's pyridine moiety to E245 in the αC helix.

Direct comparison of the binding modes of LDN-212854 and LDN-193189 is critical to provide a rational for their different selectivity (Fig. 1, Fig. 3). The overall binding poises of these molecules are almost identical. Moreover, the conformations of all side chains within the ATP-binding pocket are unchanged. Instead, differences are observed in the water networks surrounding the quinoline nitrogens (Fig. 3). In the inactive ALK2 conformation the catalytically important salt bridge between Lys235 (β3 strand) and Glu248 (αC) is broken and these side chains are free for interaction with inhibitors. The nitrogen of the 5-quinoline of LDN-212854 appears optimally positioned to form a water-mediated hydrogen bond that bifurcates to contact both Lys235 and Glu248 (Fig. 3A). The position of Lys235 is further stabilized by another water-mediated hydrogen bond to Asp241 also located within the αC helix (Fig. 3A).

By comparison, the 4-quinoline of LDN-193189 places the nitrogen deeper into the back pocket of the ATP-binding site (as also found in the 4-pyridine of dorsomorphin, Fig. 1). This nitrogen also forms a water-mediated hydrogen bond to Glu248 (Fig. 3B). However, the equivalent water is shifted some 1.54 Å further back into the ATP-binding pocket to better match the 4-quinoline. As a result, this water is moved beyond the normal hydrogen bond distance from Ly235 (3.54 Å, Fig. 3B). Thus, the 5-quinoline of LDN-212854 appears particularly favourable for establishing a larger network of water-mediated hydrogen bonding in ALK2.

### 3.5 Differences in the inhibitor binding of ALK2 and ALK5

We also wanted to compare structures of ALK2 and ALK5 to explore whether the observed water network might additionally contribute to the far weaker binding of LDN-212854 to ALK5 relative to other small molecule BMP type I receptor inhibitors. This is germane given the high sequence conservation between ALK2 and ALK5 in the relevant back pocket region. Unfortunately, there are no available ALK5 structures that are directly comparable with a bound small molecule BMP type I receptor inhibitor. Instead, we analyzed a range of representative ALK5 structures including the apo state (PDB 5E8S [50], Fig. 3C) and one bound to a pyrazolo[4,3-*b*]pyridine-based ligand (PDB 5USQ [51], Fig. 3D). Most notably, structures of the isolated kinase domain of ALK5 show a greater tendency to adopt an active conformation as evidenced by the correct positioning of the αC helix, which enables the Lys232-Glu245 salt bridge (Fig. 3C and D). Thus, in these ALK5 structures water is excluded directly between these side chains and instead other water-mediated hydrogen bond interactions are observed, such as between Ser241 and Glu245 (Fig. 3C).

**Fig. 4:**
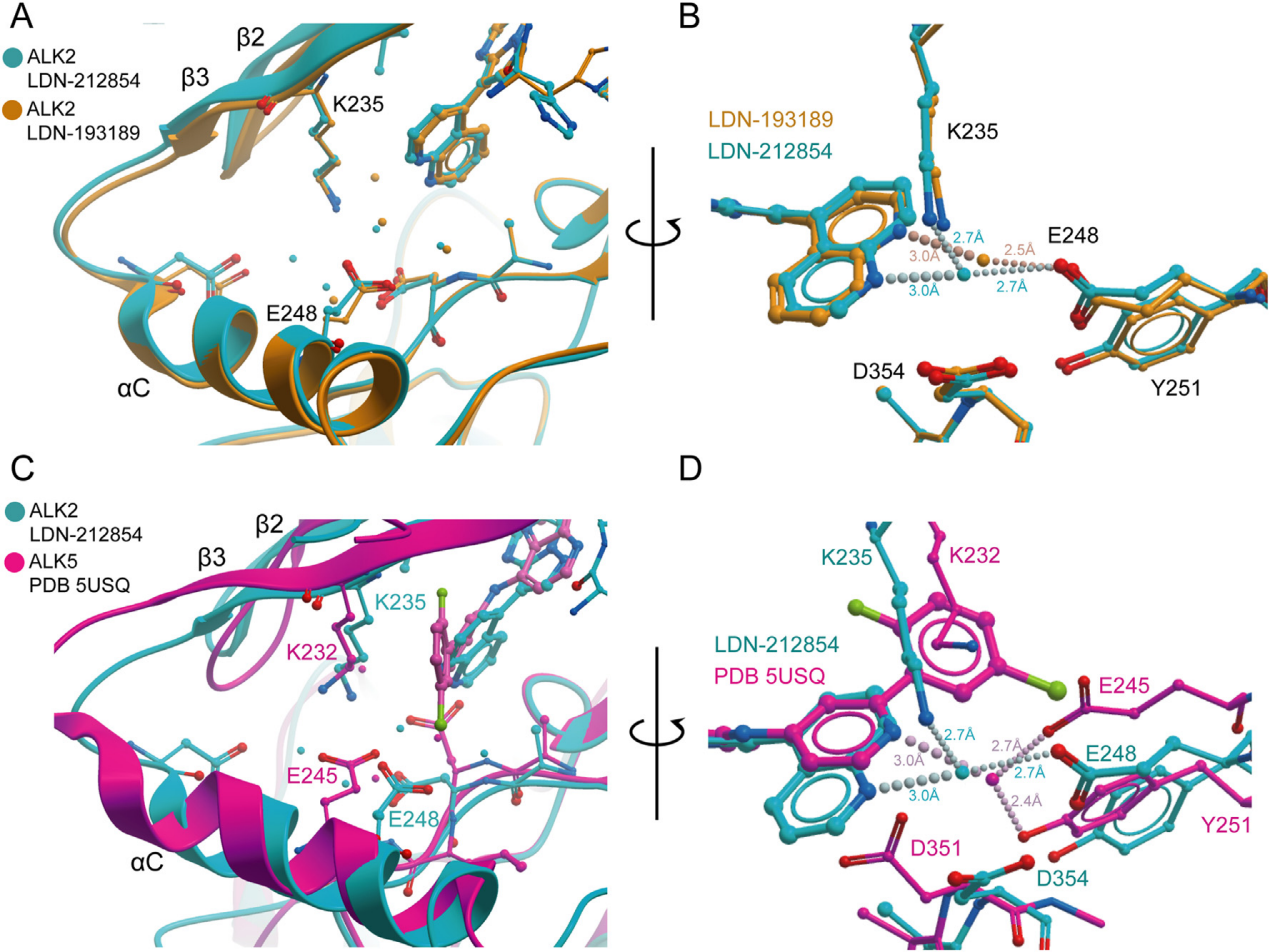
Comparison of inhibitor interactions in ALK2 and ALK5. (A) Superposition shows that the overall binding poises of LDN-193189 (orange) and LDN-212854 (blue) in ALK2 are closely conserved, but there are subtle changes in the surrounding water networks. (B) Rotated view showing a close up of the quinoline interaction. The binding of the 4-quinoline of LDN-193189 (orange) includes a key water-mediated hydrogen bond to E248. An equivalent water in the LDN-212854 co-structure (blue) is shifted slightly allowing interaction with both E248 and K235. (C) Superposition of ALK2 (blue) and ALK5 (purple) shows a conformational change that alters the position of key side chains. Waters observed in the ALK2 and ALK5 co-structures, colored blue and purple, respectively, are also positioned differently. (D) Rotated view as in panel B. Superposition shows a pyridine nitrogen in the ALK5 inhibitor that is positioned equivalently to the 4-quinoline nitrogen of LDN-193189 bound to ALK2. In the ALK5 structure (purple), this nitrogen forms a comparable water-mediated hydrogen bond to Glu245 in the αC helix, as well as an extra contact to the side chain of Y249. By comparison, the water bound to LDN-212854 in the ALK2 structure is shifted towards the front of the ATP-binding pocket.

Whereas different ALK2 structures closely match (Fig. 4A and B), the superposition of ALK2 and ALK5 structures reveals shifts in conformation and side chain position that significantly change the character of the back pocket around the site of the 5-quinoline of LDN-212854 (Fig. 4C and D). The bound ligand in the ALK5 structure corresponding to PDB 5USQ includes a pyridine moiety with a nitrogen atom positioned identically to that of the nitrogen in the 4-quinoline of LDN-193189 (Fig. 4D). In the ALK5 structure, this nitrogen forms a comparable water-mediated hydrogen bond to Glu245 in the αC helix, as well as extra contacts to the side chain of Tyr249 and to the backbone amide of Asp351 (Fig. 4D). Thus, this nitrogen position appears particularly favourable for the ALK5 structure, although extending the pyridine into a quinoline would necessarily alter the position of the Asp351 side chain to avoid a steric clash (Fig. 4D). The binding of LDN-212854 to ALK5 would necessitate a change in this water network and therefore likely destabilize the current conformation. For example, a new water position bridging between Glu245 and the 5-quinoline would likely disrupt the former water-mediated interactions with Tyr249 and Asp351.

## 4 Conclusions

**Fig. 5:**
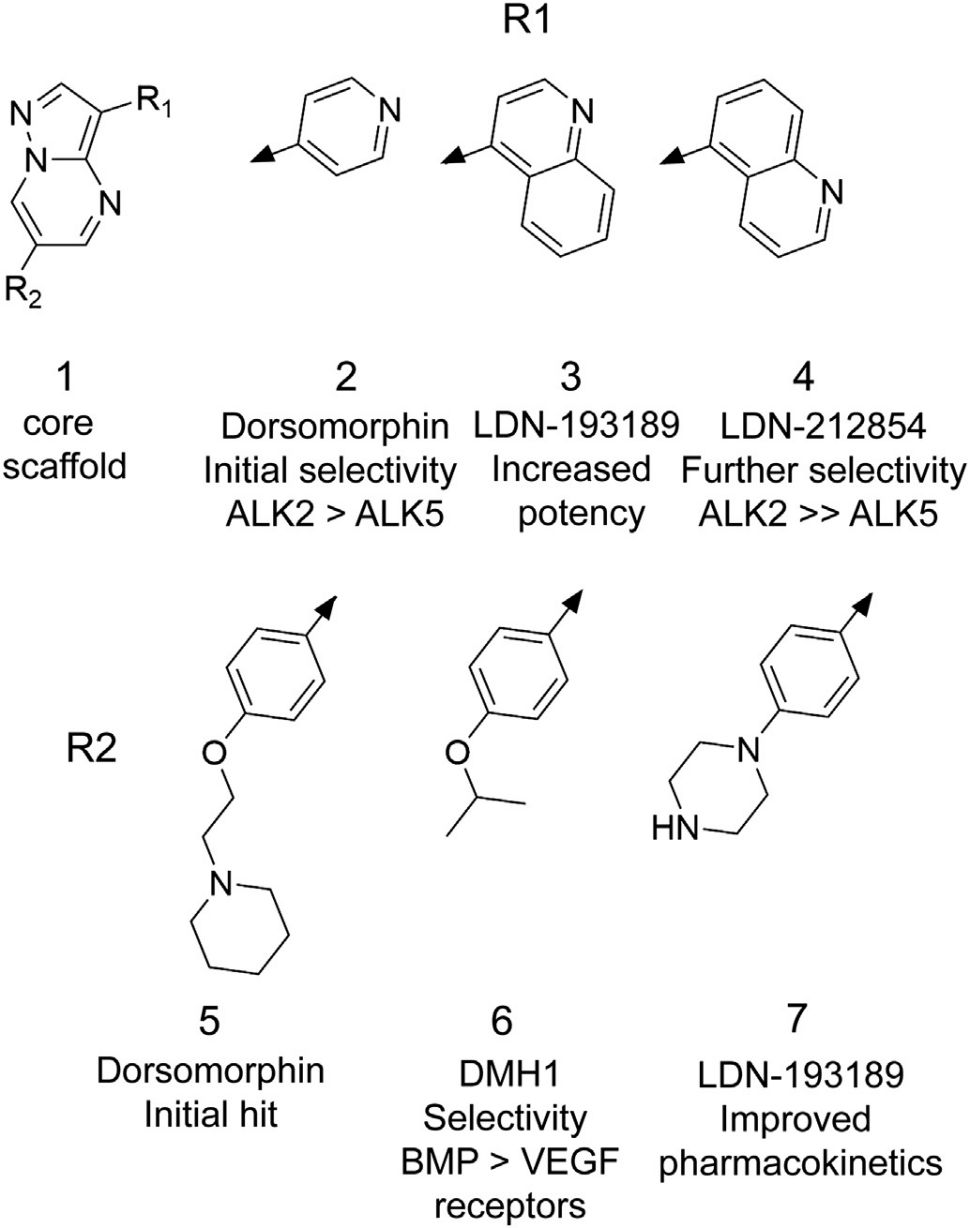
Structure-activity relationship for small molecule BMP type I receptor inhibitors. The pyrazolo[1,5-*a*]pyrimidine scaffold has been modified at the R1 and R2 positions to derive changes in potency, selectivity and pharmacokinetics.

The development of small molecule kinase inhibitors targeted specifically to BMP or TGFβ receptors has provided valuable reagents to interrogate these signalling cascades in biology [7], [28]. Derivatives of the pyrazolo[1,5-*a*]pyrimidine scaffold have proven particularly successful for the development of small molecule BMP type I receptor inhibitors, exploiting the R1 and R2 positions shown in Fig. 5 for potency, selectivity and pharmacokinetic properties. Given the variety of inhibitors now available, it is important that the most appropriate inhibitor is selected for any specific assay. LDN-193189 is suitable as a pan-BMP type I receptor inhibitor, whereas LDN-212854 is recommended for experiments specifically investigating the function of ALK2. Its inhibitory activity is highest against ligands such as BMP6 and BMP7 which favour ALK2, but decreased against ligands such as BMP2 and BMP4 which favour ALK3 [33]. Thus, careful titration of these two inhibitor molecules can enable specific insights into receptor utilization [33].

**Fig. 6:**
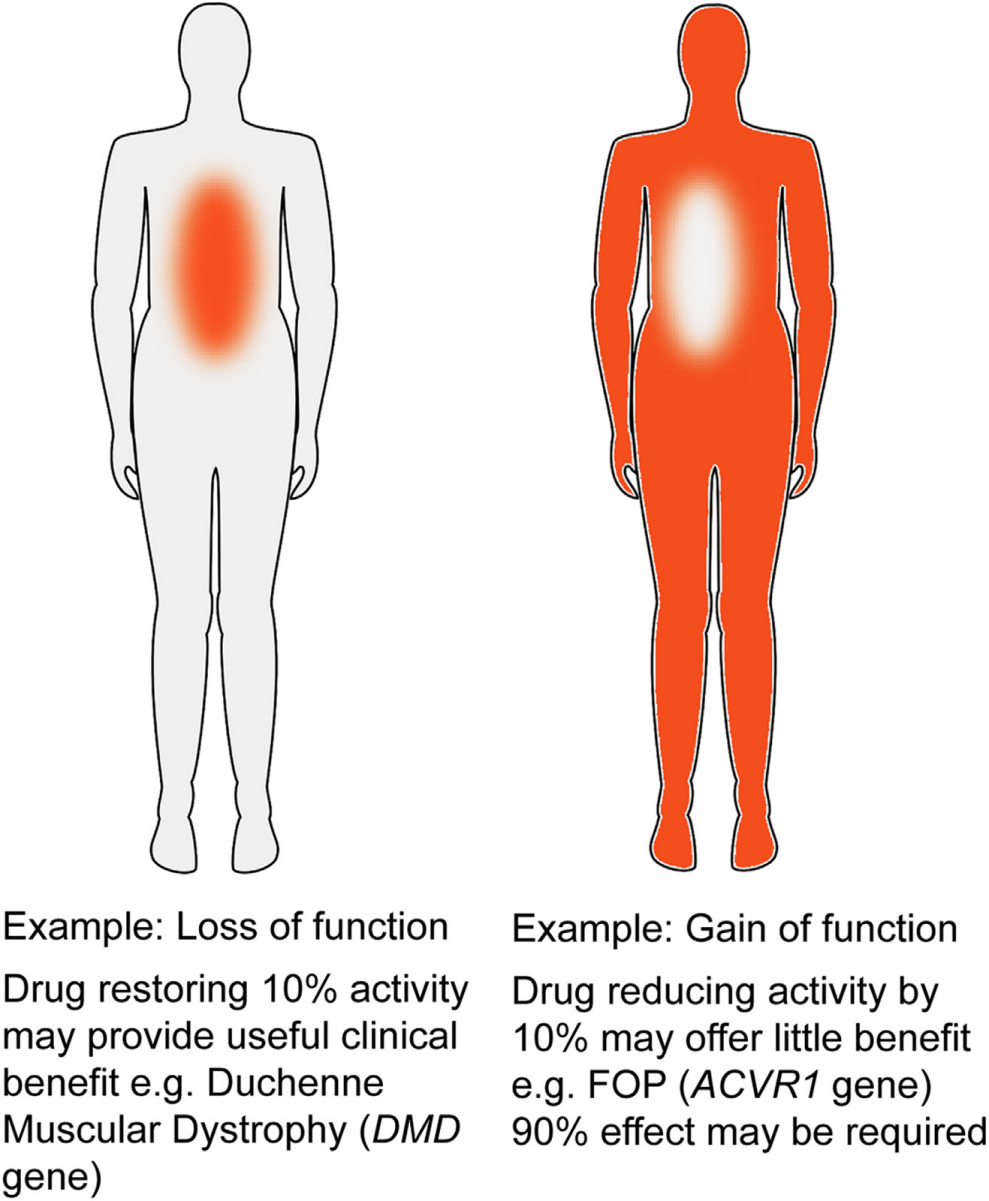
Schematic comparing the clinical requirements of drugs targeting diseases with loss and gain of function mutations, respectively.

Small molecule inhibitors targeting ALK5 have demonstrated dose limiting cardiac toxicity in the clinic [52]. Efforts to derive ALK2-selective inhibitors for therapeutic application have therefore also aimed to minimize any off-target ALK5 interaction [33], [36], [37]. Much of this work has been directed towards the rare congenital syndrome FOP, which is strictly linked to gain of function mutations in the BMP type I receptor ALK2 [11]. All patients with FOP experience episodes of heterotopic ossification in muscle, tendons and ligament that progressively restrict joint movement, such that most individuals are confined to a wheelchair by their third decade of life. The clinical challenge presented by FOP is notably distinct from other disabling pathologies such as Duchenne Muscular Dystrophy (DMD), which is associated with loss of function mutations in the dystrophin gene *DMD* [53]. In DMD, a drug restoring 10% of dystrophin function may offer significant benefit for a patient's quality of life and muscle function. By contrast, a drug reducing heterotopic ossification by 10% is expected to offer little meaningful impact for FOP patients and a 90% effect or more may be required (Fig. 6). Thus, potency and selectivity will both be critical parameters for the therapeutic index of any future ALK2 inhibitors entering into clinic trials.

In summary, the inactive conformation of the ALK2 kinase appears to be well suited to the inhibitor scaffold of LDN-212854 and supports a rich network of water-mediated hydrogen bonds. The structure of this complex provides a template for the design of future analogues as well as other chemical series that may exploit this water network to achieve similar levels of potency and selectivity.

## Conflicts of interest

None.
